# The answer to the question “What is the best housing temperature to translate mouse experiments to humans?” is: thermoneutrality

**DOI:** 10.1016/j.molmet.2019.05.006

**Published:** 2019-05-18

**Authors:** Alexander W. Fischer, Barbara Cannon, Jan Nedergaard

**Affiliations:** Department of Molecular Biosciences, The Wenner-Gren Institute, Stockholm University, Stockholm, Sweden; Department of Biochemistry and Molecular Cell Biology, University Medical Center Hamburg-Eppendorf, Hamburg, Germany; Department of Genetics and Complex Diseases, Harvard T. H. Chan School of Public Health, Boston, USA; Department of Cell Biology, Harvard Medical School, Boston, USA; Department of Molecular Biosciences, The Wenner-Gren Institute, Stockholm University, Stockholm, Sweden

Most of the energy that we use in our daily life, we use in order to remain alive; this is the price we pay for restoring order in a universe that constantly strives towards chaos. We refer to this running cost for life as our Basal Metabolic Rate (BMR). It is generally measured with a hood calorimetric system for 20–30 min while the subject is requested to be inactive, not to have eaten for some time, and to be in an environment of thermal comfort. In real life, we spend extra energy moving ourselves and things around, but, relatively speaking, on a daily basis, this is not very much; an additional ≈70–80% on top of our BMR is what most studies indicate [1] (with some variation based on the actual life conditions, and thus with the lower value (70%) attributed to North American lifestyle and the higher value (80%) attributed to European lifestyle). The ratio between the total daily Mean Energy Expenditure (MEE) and BMR is referred to as the Physical Activity Level (PAL), and it is thus normally expected to be 1.7–1.8. Indeed, 1.7 is the PAL level that was strongly advocated by Speakman and Keijer (2012) [2] to be representative of human metabolism.

Mouse studies generally attempt to mimic as closely as possible the human condition if they are to be considered relevant for translational application. If we concentrate here on metabolism, then, based on the above, it is clear that mouse housing conditions should be such that actual mouse mean metabolism/energy expenditure (MEE) should be about 1.7 times BMR. This was the starting point for this discussion, and we all agree that this should be the goal.

## 1 The original Speakman/Keijer suggestion

In 2012, Speakman and Keijer published a paper in which they arrived at the conclusion that to obtain metabolic rates of 1.7 times BMR in mice, the mice should be kept at about 23 °C [2]. This is substantially below mouse thermoneutrality, which is a zone around 28–32 °C in normal adult mice. Speakman and Keijer arrived at this conclusion by assuming that reported measurements of mouse metabolic rate at thermoneutrality (30 °C) were equal to mouse BMR. After considering a series of studies where mouse metabolism had been measured at different temperatures (Scholander plots), Speakman and Keijer found that at about 23 °C, the metabolic rate of mice was about 1.7 times the reported rate at 30 °C. From this, they concluded that 23 °C was the optimal temperature for translational experiments, at least in single-caged mice. Additionally, they discussed that the optimal temperature could even be a little lower. If other factors (e.g., huddling, nest-building) were taken into account, even standard housing temperatures (20 °C) could be considered adequate. In many ways, this was a comforting conclusion, since it meant that standard housing conditions in animal facilities for mice were fully adequate for translational metabolic studies.

## 2 We identify “true” mouse BMR

In 2018 [3], we demonstrated experimentally that the assumption made by Speakman and Keijer that mouse BMR was equal to mouse metabolic rate at 30 °C was not feasible. Indeed, using high time-resolution calorimetry, we established that mice at 30 °C showed considerable variation in their metabolic rates. In parallel to what is the case in humans, this variation consists of true BMR plus varying levels of PAL. Since it is not possible to instruct a mouse to remain inactive, and as the mouse may be upset if it does not receive anything to eat, identification of the true BMR of the mouse at 30 °C in the same way as is done for humans is not feasible; a pragmatic definition has to be made. We chose to identify an interval of 5 *consecutive* 2-minute measurements during which the mouse had the lowest metabolic rate as being the best approximation of true BMR. We found that in the individual mice, these 10-minute intervals of minimal metabolic rate all occurred during late afternoon, i.e. a time when the mice are resting and not eating. This consistency of timing of the estimated BMR implies that our pragmatic definition of BMR is of biological relevance.

Importantly, we found that when we divided the daily mean energy expenditure (MEE) of the mice at 30 °C with the BMR defined in this way, we obtained a ratio of 1.8 – i.e. the same ratio between BMR and daily energy expenditure as is seen in normal human populations. The reason that this had been overlooked previously was probably that common metabolic measurements use a low time-resolution. Since the daily mean energy expenditure is increased when mice are living below thermoneutrality, we concluded that mice housed at any temperature below thermoneutrality (i.e. below what is referred to as the “lower critical temperature”) will be under conditions where their daily metabolic rate is more than 1.8 times their BMR. Therefore, we concluded that housing a mouse at any temperature below its lower critical temperature (i.e. below thermoneutrality) would result in PALs that exceeded 1.8, and this would not be a humanized environmental condition for mice. Only at thermoneutrality would mice show human behavior in this respect.

## 3 The updated suggestion by Keijer, Li, and Speakman

**Figure 1:**
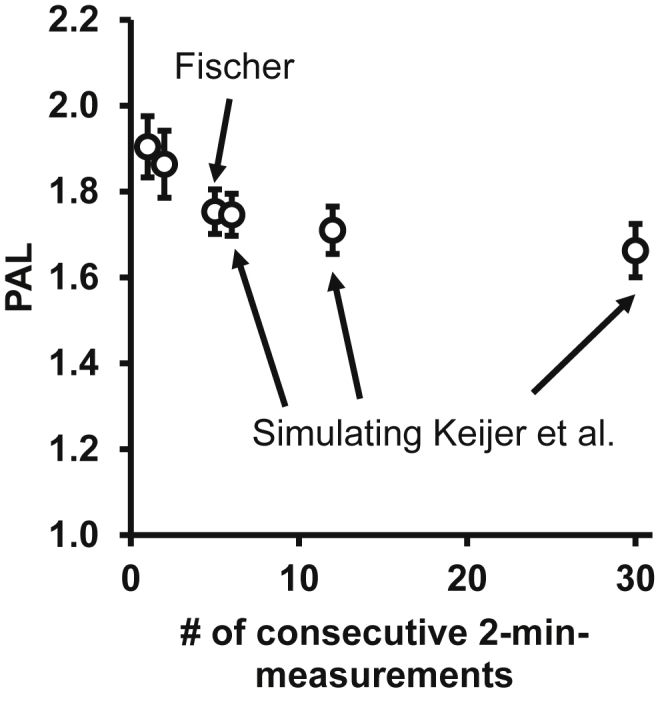
Effect of time resolution on estimated PAL ratios. Physical activity level (PAL) values are the daily mean energy expenditure divided by the basal metabolic rate (BMR). To obtain the BMR, an estimate of the lowest energy expenditure must be arrived at. The length of time on which the BMR is based will affect the outcome. This time has to be balanced between erratic low oxygen consumptions of short duration and the effect of activity occurring during longer time intervals. Based on the data presented earlier [3], we calculate here the effect of the length of the sampling time on the PAL. The data consist of the 2-minute readings we obtained in our high time-resolution calorimetry system (i.e. we did not use “short transient metabolic declines” as we are incorrectly cited as doing by Keijer et al. [4]). As seen, using only one 2-minute lowest reading or 2 successive 2-minute readings will lead to probably somewhat unrepresentative estimates of BMR and thus of PAL. However, the sampling of 5 successive lowest readings puts the value on the extrapolation of the general trend that indicates the effect of an increasing fraction of activity on the estimate. The data from 6 points (12 min) correspond principally to the highest time-resolution of Keijer et al.; the other arrows correspond to their 24- and 60-minutes’ resolution. However, one reading with a time resolution of 12 min is mathematically probably somewhat higher than the reading of 6 lowest consecutive 2-minute intervals. Due to this, the BMR will be somewhat overestimated and the PAL somewhat underestimated. This is probably the explanation for the small and principally unimportant differences in PAL estimates that still exist between Fischer et al. (2018) [3] and Keijer et al. (2019) [4].

In 2019, we are pleased to find that Keijer and colleagues [4] have experimentally arrived at exactly the same result that we had. In a world that often discusses experimental irreproducibility, it is gratifying that studies performed in different laboratories, with different experimenters and different apparatus and at different times, actually arrive at the same results. Keijer et al. were unable technically to increase time resolution to less than 12 min, but this is close to the 10 min we had elected to use (see Figure 1 for details). Indeed, when they did this, Keijer et al. arrived at the same result as we did, that the daily metabolic rate at 30 °C is 1.7 times higher than the BMR defined as the lowest 12 min reading (which, also gratifyingly, also occurred in their mice systematically in the late afternoon). Thus, experimentally, we cannot be more in agreement.

Thus, since Keijer et al. and we arrived experimentally at the same value (1.7–1.8), and since this also agreed with the value (1.7) in the earlier paper from Speakman and Keijer, this would appear to indicate that Keijer et al. would concur with our conclusion that thermoneutrality is the optimal housing temperature for mouse metabolic studies directed to being translatable into humans. However, rather surprisingly, Keijer et al. [4] challenge our earlier results. Since we obtain the same results as they have, this criticism is in itself difficult to rationalize. Keijer et al. argue at length against the use of short instantaneous measurements as estimates of BMR. We could not agree more. As is very clearly stated in our experimental paper, we used 10-minute intervals, exactly as suggested by Speakman (2013) [5], to avoid the confounding effects of short, strong reductions in metabolism that, as also discussed by Keijer et al. [4], are probably unrepresentative states of apnea. Since this discussion about short instantaneous measurements appears to be the only argument against our data and since Keijer et al. have obtained exactly the same results as we did, we can only unite in a common realization of reality.

**Figure 2:**
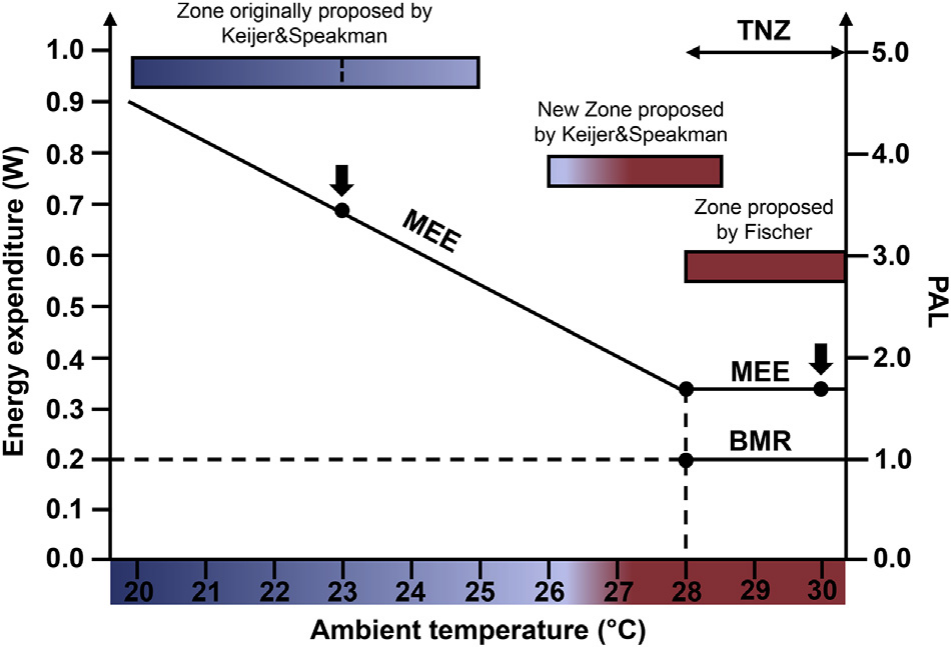
Overview of housing temperatures discussed here. In this plot, the BMR (basal metabolic rate) indicated is that measured at 30 °C by Fischer et al., 2018 [3] as 0.20 W. The diurnal Mean Energy Expenditure (MEE) at 30 °C measured by Fischer et al., 2018 [3] is 0.36 W. The ratio between these (PAL) is 1.8. The ratio MEE/BMR (PAL) measured at 30 °C by Keijer et al., 2019 [4] is 1.66 (nearly within the size of the lines). The temperature of 30 °C (right black arrow) was chosen as representative for a temperature within the thermoneutral zone (TNZ) that – e.g. according to the Keijer et al., 2019 [4] article, extends down to 28 °C. A housing temperature of 23 °C (left black arrow) was the one originally recommended by Speakman and Keijer (2013) [2] because the mean energy expenditure at this temperature was 1.7 times the mean energy expenditure (MEE) at thermoneutrality. In the Discussion section in that paper, Speakman and Keijer further suggested that the acceptable housing temperature zone could be extended nearly down to 20, provided there was provision of sufficient nesting material and that huddling could occur (indicated as the left part of the box in the figure). This temperature corresponds to a MEE of 1 W, i.e. a PAL value of 5. However, in the more recent paper from Keijer, Li, and Speakman [4], the authors now recommend a temperature of 27.2–29.1, based on their 12-minute BMR measurements, with the higher temperature being the one corresponding to a PAL of 1.7 (the North American value). This zone thus overlaps with thermoneutrality. Therefore, the present Keijer et al. paper and our earlier paper (Fischer et al., 2018) [3] fully concur in the conclusion that thermoneutrality is the optimal housing temperature, provided that the metabolic criterion is a PAL of about 1.7.

As a consequence of the concordance of our experimental results, the conclusion of the new paper of Keijer et al. is that their recommended temperature has risen from 23 °C (according to the earlier Speakman and Keijer paper [2]) to 25.5 °C–27.6 °C and even up to 29.1. This latter temperature (that is stated by Keijer et al. as the best housing temperature to mimic North American human metabolism) is actually above the lower limit for the thermoneutral zone, defined here by Keijer et al. [4] as 28 °C. We are thus in complete agreement: to best mimic human metabolism, housing temperatures should be within the thermoneutral zone, i.e. around 28–30 °C (see Figure 2 for a visual summary).

## 4 What then in reality is the best housing temperature?

Keijer et al. [4] also present an extensive discussion in which they appear to promote an idea that sometimes the best humanized condition is thermoneutrality, but, other times, it could be “standard” conditions (20 °C) or perhaps their earlier suggested intermediate temperatures. This is because some “responses” that are observable at 20 °C become invisible at 30 °C; thus, pragmatically, 20 °C could sometimes be the preferred temperature. Here we would strongly advocate the opposite a-priori opinion: it is often directly misleading to use temperatures below thermoneutrality in studies intended to translate to human metabolism.

We are most familiar with these problems in relation to the recruitment process in brown adipose tissue. In particular, there are studies published in which certain food components or other agents (e.g., gene mutations) are suggested to cause “browning,” in that UCP1 gene expression is increased in the treated mice. This is associated with increased metabolism and slimming, or protection against obesity. These experiments are generally performed under normal housing conditions, i.e. at about 20 °C. However, as we have discussed in more detail elsewhere [6], the action of several of these agents is to cause insulation problems (the mice lose fur or the fur changes structure) or to induce vasodilation. This means that the mice feel colder at any temperature below thermoneutrality, and the recruitment of brown/beige adipose tissue is secondary to the increased heat loss. In contrast to what these papers imply, the agents, therefore, are not enhancers of metabolism but inducers of heat loss; thus, they are not potentially translatable as human slimming agents. At thermoneutrality, the brown-fat recruitment disappears. Thus, this is one example of the risk of using standard housing temperatures; they may yield false positive results. Worse, however, is that the high metabolic rate caused by the relative cold at 20 °C can overshadow potentially positive effects of “agents” on, for example, food intake, which could have been observable at thermoneutrality, during which food consumption is not governed by a cold-induced enhancement of food intake. This means that the use of standard housing conditions can also lead to false negatives: that agents that actually could positively affect metabolism remain unrecognized.

We believe that similar issues may be relevant in many other types of translationally directed studies. Thus, if the effect of an agent disappears if tested at thermoneutrality, there is good reason to be cautious about further promoting it translationally. An even better experimental adjustment would be to always perform metabolic (and other translationally directed) studies at thermoneutrality.

## 5 It is thermoneutrality that we advocate

Finally, let us also point out that what we advocate is thermoneutrality – not 30 °C. This temperature is merely a convenient setting for actual housing temperatures for thermoneutrality in normal adult mice, within a thermoneutral zone of about 28–32 °C. However, this temperature is not necessarily thermoneutral under all conditions. For example, pregnant [7] and lactating [8] animals continuously generate extra heat, and they would therefore have a much lower thermoneutral zone.; There is therefore good reason to breed mice at lower temperatures than 30 °C, i.e. at temperatures that are thermoneutral to pregnant and lactating mice. Presently, direct experimental data on the thermoneutral zones of pregnant and lactating mice, however, seem not to be available, but standard housing conditions of about 20 °C are probably better for the dams than about 30 °C [9].

As to the final conclusion, we will again point out that the data from both groups and the practical consequences of the data are now in agreement. Thus, in reality, we all now conclude [3], [4] that to obtain PAL values around 1.7 and thus to mimic the metabolic status of humans, mice should be studied at temperatures within their thermoneutral zone.

## Conflicts of interest

None.
